# Quality of life among Indian paediatric patients suffering from constipation: An evaluative study

**DOI:** 10.6026/973206300221310

**Published:** 2026-03-31

**Authors:** Alka Gupta Grewal, Shashi Mauli Singh, Nitish Kumar, Urvashi Raj, Abraham George, Saira George, Rahul Tiwari, Heena Dixit

**Affiliations:** 1 Department of General Surgery, ESIC Medical College & Hospital, Ludhiana, Punjab, India; 2 Department of Pediatrics, FH Medical College, Etmadpur, Agra, Uttar Pradesh, India; 3 Department of Pedodontics, Yamuna Institute of Dental Sciences and Research, Yamuna Nagar, Haryana, India; 4 Department of Orthodontics, St Gregorios Dental College, Cheladu, Kerala, India; 5 Department of Conservative Dentistry and Endodontics, St Gregorios Dental College, Chelad, Kerala, India; 6 Department of Oral and Maxillofacial Surgery, Narsinhbhai Patel Dental College and Hospital, Sankalchand Patel University, Visnagar, Gujarat, India; 7 Department of Medical Health Administration, Index Institute, Malwanchal University, Index City, Nemawar Road, Indore, Madhya Pradesh, India

**Keywords:** Paediatrics, functional constipation (FC), health-related quality of life (HRQoL), pediatric quality of life inventory (PedsQL), Rome IV

## Abstract

Paediatric functional constipation is a common functional gastrointestinal disorder that significantly impairs health-related quality of life (HRQoL), yet its psychosocial and school-related impact remains under-recognized in routine clinical assessment. Therefore, it is of interest to evaluate the health-related quality of living (HRQoL) in children with functional constipation by using verified child and parent-reported tools and compared results to healthy controls that were age-matched. A cross-sectional, clinic-based sample was analyzed using standard scoring methods and inferential statistical data to compare groups along with severity and intensity gradients. Constipation- related children showed significant lower total HRQoL and psychosocial scores as well as greater symptom burden scores than controls and had the lowest HRQoL being observed in those who suffer from frequent painful defecation as well as fecal incontinence. Thus, we show the importance of routine HRQoL assessments in conjunction with symptom evaluation to provide a comprehensive treatment plan and monitor the response to behavioral and pharmacologic treatments.

## Background:

Functional constipation (FC) is one of the most frequent functional gastrointestinal disorders (FGIDs) in children and experts a significant impact on outpatient clinics and caregiver worry. Current epidemiological evidence suggests that FGIDs are common already in early childhood and functional constipation (FC) becomes a predominant condition during the toddler and preschool period [1]. The Rome IV framework encourages a symptom-based clinical diagnosis (including fecal retention) and research studies of diagnostic performance show that structured criteria/questionnaires can assist in standardizing assessment as well as highlighting the limitations of a self- report in younger children [2, 3-4]. In addition to increased bowel frequency, FC is frequently associated with painful defecation, faecal impaction and pain, abdominal pain as well as at times incontinence of stool that can last for months or even longer due to the development of avoidance behaviours [3, 4-5]. Additionally, FC is not just a bowel habit disorder but it has an impact on school participation, sleep, appetite, mood and overall family routines and perceived well-being. Reviews during the Rome IV era highlight that FC causes measurable HRQoL impact on children and caregivers, with chronicity of symptoms and refractoriness to treatment increase psychosocial burden [5]. Therefore, it is of interest to describe and compare the health-related quality of life in children with functional constipation and age-matched healthy controls using validated assessment instruments.

## Materials and Methods:

## Study design and setting:

Cross-sectional evaluative study conducted in a paediatric gastroenterology outpatient clinic (tertiary care).

## Participants:

Children aged 5-16 years meeting Rome IV-compatible clinical diagnosis of functional constipation were enrolled consecutively. A comparison group of age- and sex-matched healthy controls was recruited from general paediatric clinics and community health visits. Exclusion criteria included known organic gastrointestinal disease, neurologic disorders affecting bowel function, Hirschsprung disease, endocrine disorders, chronic systemic illness and recent gastrointestinal surgery.

## Measures:

HRQoL was assessed using the pediatric quality of life inventory (PedsQL) generic core scale (child self-report for ≥8 years and parent-proxy where appropriate) and a constipation-related symptom/impact measure aligned with contemporary paediatric constipation assessment tools and psychometric approaches [6, 7-8]. For cross-cultural settings, validated adaptations of constipation scoring or constipation-related questionnaires were preferred to ensure reliability and interpretability [6, 7]. Clinical severity was operationalized using stool frequency, painful defecation, fecal incontinence episodes and need for disimpaction/ongoing laxatives.

## Statistical analysis:

Continuous variables were summarized as mean ± SD; categorical variables as n (%). Group comparisons used independent-samples t- test or Mann-Whitney U test based on distribution; categorical comparisons used chi-square test. One-way ANOVA tested HRQoL across constipation severity strata. Statistical significance was set at p<0.05.

## Results:

A total of 240 children were analysed: 120 functional constipation and 120 controls (mean age 10.2 ± 2.8 years; 55% male). Within the constipation group, median symptom duration was 10 months (IQR 6-18). Painful defecation (≥1/week) was reported in 74%, fecal incontinence in 28% and prior disimpaction in 32%. Controls reported no chronic bowel complaints and no prior laxative use. Children with functional constipation and controls were comparable in age, sex distribution and BMI (all p>0.05), indicating reasonable group matching. In contrast, constipation-specific clinical burden was markedly higher in the constipation cohort: nearly three-quarters reported painful defecation at least weekly and more than one-quarter reported fecal incontinence. Ongoing laxative use was common among constipation cases and absent in controls. These differences confirm a clinically distinct constipation phenotype suitable for HRQoL discrimination analyses (Table 1 - see PDF). Across the constipation cohort, PedsQL Total Score was lower than controls, with the largest deficits in psychosocial health (emotional, social and school functioning). Symptom-burden scores (constipation impact measure) were higher in constipation cases and correlated moderately with reduced HRQoL total score (Pearson r ≈ -0.46, p<0.001). Severity stratification showed a monotonic decline in HRQoL from mild to severe constipation, particularly among children with fecal incontinence and frequent painful defecation (Table 2 - see PDF).

## Discussion:

The study evaluative supports the notion that functional constipation in children has been associated with clinically significant problems with HRQoL, specifically in the domains of psychosocial and school. The observed pattern is consistent with the Rome IV-era epidemiology, revealing an elevated FGID prevalence in children of early age and the importance of constipation as a significant cause of paediatric symptom burden [1]. Standardization of diagnostics within Rome IV remains important; however, there is evidence to warn against relying solely on self-reported questionnaires, without ensuring understanding and proper administration, especially in children younger than 2 years old [2, 3- 4]. These considerations for diagnosing are important because impairment in HRQoL is not limited to frequency of bowel movements but also by perception of pain and anxiety associated with toileting and the family's response to symptoms. These factors can be missed when the assessment is only symptom-based. The most important implication is that HRQoL needs to be integrated in routine constipation care routes. Recent studies emphasize constipation as a condition with biopsychosocial factors and significant effects on the child's well-being as well as family function [5, 9]. Additionally, the work of psychometrics in the field of paediatric constipation has improved over the past few years. This includes with validated questionnaires for constipation and cross-cultural adaptations that increase the validity of severity-grading and outcomes assessment in a variety of environments [6, 7-8]. This is particularly important for centers that serve multilingual populations and where tools validated according to cultural norms can reduce measurement errors and allow for better monitoring. The management advancements in the period 2020-2025 also highlight the importance of HRQoL's endpoints. Pharmaceutical updates continue to support polyethylene glycol-based strategies in conjunction with behavioral interventions and acknowledge that therapy-resistant constipation is a significant clinical subgroup [10, 11, 12, 13, 14-15]. The development of telemedicine-based bowel management program and other mobile services reflects efforts to increase the adherence, self-efficacy, as well as engagement, which are directly linked to HRQoL, not stool metrics by themselves [11, 13]. In addition, data from the national scale of epidemiology show that medically treated constipation is still a major and, possibly, growing health care burden which is proving the importance of outcomes that reflect health status and quality of life [12]. In parallel, guideline-development initiatives (including joint ESPGHAN/NASPGHAN protocols) signal the field's intent to refine evidence- based pathways and outcomes selection, creating opportunity to standardize HRQoL inclusion across studies and routine care [14]. Clinically speaking, the greatest problems with HRQoL in school and psychosocial functioning suggest that treatment for constipation should include anticipatory guidance as well as school support plans and screening for avoidance and distress behavior. The future work should focus on studies that examine whether improvement in HRQoL is correlated with symptoms control over time and to determine which interventions are most effective to help to restore psychosocial functioning, specifically for children suffering from fecal incontinence or a long duration of symptom.

## Conclusion:

Paediatric functional constipation can be linked to significant decreases in HRQoL and a significant mental and school-related impairment. Regular integration of confirmed HRQoL measurements can improve the clinical evaluation beyond stool outcomes and assist in the development of holistic treatments. The future guideline-aligned care pathway should establish a standard for HRQoL as a primary outcome that reflects the benefits that are centered on families and patients.

## Advancement to knowledge:

This study adds contemporary, Rome IV-aligned evidence demonstrating that paediatric functional constipation is associated with significant and graded impairment in health-related quality of life, particularly in psychosocial and school domains, thereby supporting the routine integration of validated HRQoL measures into clinical assessment and outcome monitoring.
